# Antioxidant and Antityrosinase Activities of Various Extracts from the Fruiting Bodies of *Lentinus lepideus*

**DOI:** 10.3390/molecules16032334

**Published:** 2011-03-10

**Authors:** Ki Nam Yoon, Nuhu Alam, Kyung Rim Lee, Pyung Gyun Shin, Jong Chun Cheong, Young Bok Yoo, Tae Soo Lee

**Affiliations:** 1 Division of Life Sciences, University of Incheon, Incheon 406-840, Korea; 2 Mushroom Division, National Institute of Horticultural & Herbal Science, RDA, Suwon 441-707, Korea

**Keywords:** antioxidant, *Lentinus lepideus*, phenolic compounds, tyrosinase inhibition, xanthine oxidase

## Abstract

*Lentinus lepideus* is an edible mushroom currently available in Korea. The acetone, methanol and hot water extracts were prepared and assayed for their antioxidant and antityrosinase inhibitory activities. The hot water extract showed the strongest β-carotene-linoleic acid inhibition compared to the other extracts. At 8 mg/mL, the methanolic extract showed a high reducing power of 1.21. The acetone and methanol extracts were more effective in scavenging DPPH radicals than the hot water extract. The strongest chelating effect was obtained from the methanolic extract. Xanthine oxidase and tyrosinase inhibitory activities of the acetonic, methanol and hot water extracts increased with increasing concentration. Gallic acid, chlorogenic acid, vanillin, naringin, naringenin, formononetin, and biochanin-A were detected in the acetonitrile and hydrochloric acid (5:1) solvent extract. This study suggests that fruiting bodies of *L. lepideus* can potentially be used as a readily accessible source of natural antioxidants.

## 1. Introduction 

*Lentinus lepideus*, commonly known as the train wrecker mushroom, belongs to the family Tricholomaceteae, order Agaricales [1]. It grows on the wood of pine and other conifers, and seems to prefer railroad ties, bridge timber, old logs and stumps. It is one of the popular edible mushrooms in China and Japan, and recently this mushroom is been successfully cultivated and is commercially available in Korea.

Traditionally, mushrooms have been used in Asia as foods and medicines. Generally, mushrooms are rich in dietary fiber, minerals, vitamins and low in fat and calories [2]. Mushrooms also contain various polyphenolic and flavonoid compounds recognized as good antioxidants [3]. Moreover, several important compounds including polysaccharides (β-glucan), ergosterol, vitamins, α-tocopherol and β-carotene have been isolated from the mushrooms. Recent studies have shown that medicinal properties of mushrooms include anti-tumor, antimicrobial, liver function improving, and blood pressure and cholesterol lowering activities [4].

It was reported that the antioxidant activity of plant materials correlates well with their phenolic compound contents [5]. Phenolics are one of the major groups of nonessential dietary components that have been associated with the inhibition of atherosclerosis and cancer [6], so it is an important to consider the effect of the total phenolic content on the antioxidant activity of mushroom extracts. The bioactivity of phenolics may be related to their ability to chelate metals, inhibit lipoxygenase and scavenge free radicals. Allopurinol is a clinically used xanthine oxidase inhibitor, which suffers from many side effects such as hypersensitivity syndrome, and renal toxicity. Thus, there is a need to develop compounds with xanthine oxidase inhibitory activities which are devoid of the undesirable side effects of allopurinol. Wild edible mushrooms represent a potential source of such compounds [7]. Flavonoids and polyphenolic crude extracts have been reported to possess xanthine oxidase inhibitory activity [8].

Tyrosinase is a copper-containing enzyme present in mushroom, plant and animal tissues that catalyzes the production of melanin and other pigments from tyrosine by oxidation [9]. Despite the clinical importance of *L. lepideus* or its therapeutic potential, there have not been many studies on physiologically beneficial components. However, the antioxidant properties of this mushroom are not available. Therefore, the purpose of this study is to evaluate the antioxidant potentials and tyrosinase inhibitory activities of acetone, methanolic, and hot water extracts of the fruiting bodies of *L. lepideus*. The phenolic compound profiles were also determined.

## 2. Results and Discussion 

### 2.1. Antioxidant activity on β-carotene-linoleic acid 

Polyunsaturated fatty acids, such as linoleic acid, are easily oxidized by the oxygen in the air. This auto-oxidation leads to the occurrence of chain reactions with the formation of coupled double bonds, and at a later stage also obtaining secondary products, such as aldehydes, ketones, and alcohols. Using the β-carotene-linoleic acid method, acetone, methanolic and hot water extracts of *L. lepideus* showed different antioxidant activity patterns. The hot water extract showed the strongest linoleic acid inhibition capacity (96.32%) at 8 mg/mL, which is higher than that of the synthetic antioxidant BHT (95.21%) and TOC (96.02%), respectively at 0.5 mg/mL (Table 1).

It is probable that the antioxidative components in the mushroom extracts can reduce the extent of β-carotene destruction by neutralizing the linoleate free radical and other free radicals formed in the system. Barros *et al.* [10] reported that antioxidant activities of various extracts of *Leucopaxillus giganteus*, *Sarcodon imbricatus* and *Agaricus arvensis* increased with increasing concentration. Their antioxidant activities were 61.4, 54.3, and 46.7% at 5 mg/mL, while antioxidant activity of tertiary butylhydroquinone standard reached 82.2% at 2 mg/mL. It seems that the antioxidant activity of *L*. *lepideus* fruiting bodies was more effective than those mentioned above.

### 2.2. Reducing power 

The reducing power of acetone, methanol and hot water extracts of *L. lepideus* as a function of their concentration is shown in Table 2. The reducing power increased with increasing concentration. At 8 mg/mL, the strongest reducing power inhibition was determined in the methanolic extract with a value of 1.21 and the lowest reducing power inhibition (1.10) was exhibited by the hot water extract. Reducing power of BHT and TOC at 1.0 mg/mL were 3.21and 2.16, respectively (Table 2).

With regard to hot water extracts, the reducing power of *Hypsizygus marmoreus* was 0.99 at 5 mg/mL whereas *Agricus bisporus*, *Pleurotus eryngii*, *Pleurotus ferulae* and *Pleurotus ostreatus* showed reducing powers of 0.76, 0.75, 0.70, and 0.61 at 20 mg/mL, respectively [11]. It can be seen that the reducing power of *L*. *lepideus* was slightly lower than that of *H*. *marmoreus* and higher than those of *A*. *bisporus*, *P*. *eryngii*, *P*. *ferulae* and *P*. *ostreatus*. It was reported that the reducing power properties are generally associated with the presence of reductones, which have been shown to exert antioxidant action by breaking the free radical chains by donating a hydrogen atom [10,12].

### 2.3. Scavenging effect on DPPH

**Figure 1:**
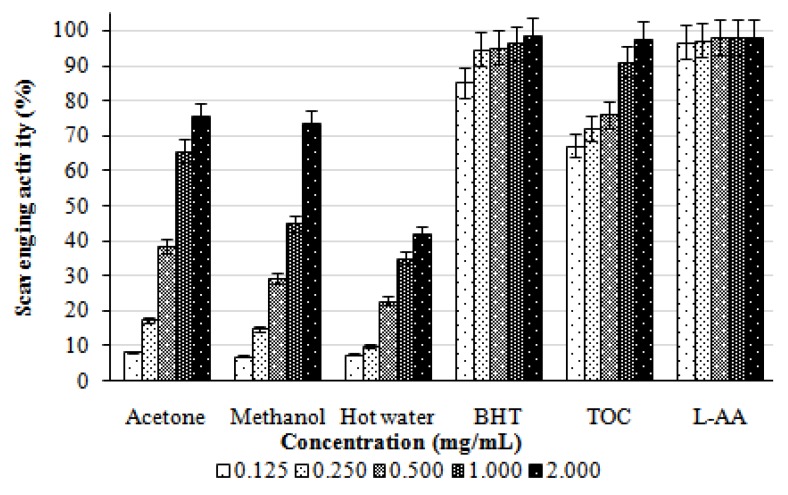
Scavenging activity of various extracts from the fruiting bodies of *Lentinus lepideus* against 1,1-diphenyl-2-picrylhydrazyl. Values expressed as means ± SD (n = 3); BHT, butylated hydroxytoluene; TOC, α-tocopherol; L-AA, L-ascorbic acid.

The scavenging effects of acetone, methanol, and hot water extracts from the fruiting bodies of *L. lepideus* on DPPH radicals increased with increasing concentration. At 0.125–2.0 mg/mL, the scavenging activities of the acetone, methanol, and hot water extracts of *L*. *lepideus* on DPPH radical ranged from 8.29–75.72, 7.20–73.80, and 7.68–42.25%, respectively (Figure 1). The results indicated that the acetone, methanol, and hot water extracts, showed good, moderate, and poor activities, respectively, at the concentrations tested. However, at 0.125–2.0 mg/mL, BHA, TOC, and L-ascorbic acid showed the excellent scavenging activities of 85.25–98.74, 67.37–97.78, and 96.74–98.23%, respectively. 

The scavenging activities on DPPH radicals by ethanolic extracts of *H. marmoreus*, *A. bisporus* and *Pleurotus citrinopileatus* fruiting bodies were 46.6–68.4% at 5 mg/mL [11]. For cold and hot water extracts at 20 mg/mL, the scavenging activities of fruiting bodies, mycelia and filtrate were 20.7–52.3, 37.6–48.3, and 19.6–23.3%, respectively. It seems that the scavenging activity of *L*. *lepideus* fruiting bodies was more effective than those mentioned above. Various extracts might react with free radicals, particularly the peroxy radicals, which are the major propagators of the autoxidation chain of fat, thereby terminating the chain reaction. Antioxidant activity of natural antioxidants has been shown to be involved in termination of free radical reaction [12]. Furthermore, Herraiz *et al.* [13] found that an essential amino acid L-tryptophan could react with phenolic aldehydes in food to form phenolic tetrahydro-β-carboline alkaloids that scavenged 2,2-azinobis (3-ethylbenzothiazoline)-6-sulfonic acid effectively. Therefore, the presence of L-tryptophan in various extracts might most likely account for the scavenging activity on DPPH radicals. However, the better scavenging activity of the acetone extract might be due to more hydrogen-donating components contained within the extracts.

### 2.4. Chelating effects on ferrous ions

**Figure 2:**
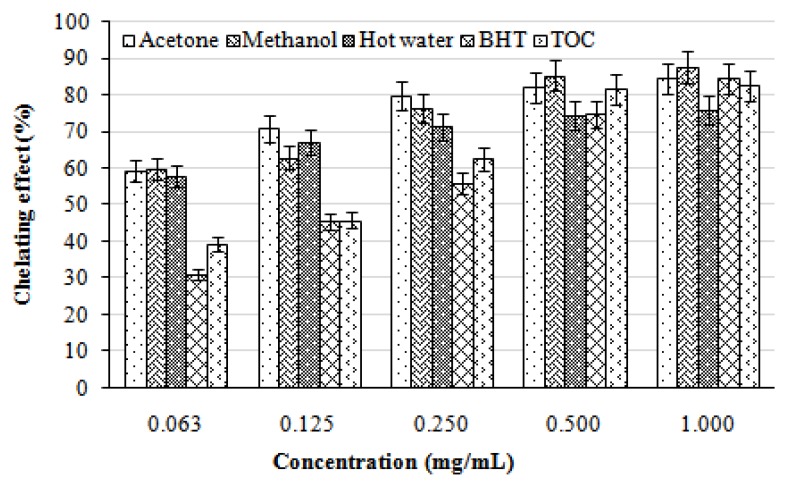
Chelating effect of various extracts from the fruiting bodies of *Lentinus lepideus*. Values expressed as means ± SD (n = 3); BHT, butylated hydroxytoluene; TOC, α-tocopherol.

In the present study, the chelating activity of the acetone, methanol, and hot water extracts from the fruiting bodies of *L. lepideus* at five different concentrations (0.063, 0.125, 0.250, 0.500, and 1.000 mg/mL) toward ferrous ions was investigated. BHT and TOC were used as reference standards on ferrous ions. As can be seen from Figure 2, the chelating capacity of the extracts increased with increasing concentration. The strongest chelating effect (87.50%) was obtained with the methanolic extracts at 1.0 mg/mL. At this concentration, the lowest chelating effect (75.91%) was exhibited by the hot water extract. All of the extracts evaluated here showed significantly higher chelating effects on ferrous ions than those of the standards, BHT and TOC, at the concentration of 0.063, 0.125, and 0.250 mg/mL, respectively. 

With regard to hot water extracts at 20 mg/mL, *Ganoderma tsugae* and *Agrocybe cylindracea* chelated ferrous ions by 42.6 and 45.8%, respectively [14]. At 1–5 mg/mL, chelating abilities of *H*. *marmoreus* and *P*. *citrinopileatus* were 75.6–92.6% [15]. It seems that chelating ability of *L. lepideus* on ferrous ions was similar to that of *H. marmoreus* and *P. citrinopileatus*, but more effective than those of *G. tsugae* and *A. cylindracea*. Chelating agents may serve as secondary antioxidants because they reduce the redox potential thereby stabilizing the oxidized form of the metal ions. Since ferrous ions were the most effective pro-oxidants in food system [16], the high ferrous-ion chelating abilities of the various extracts from the fruiting bodies of *L. lepideus* would be beneficial.

### 2.5. Analysis of phenolic compound

**Figure 3:**
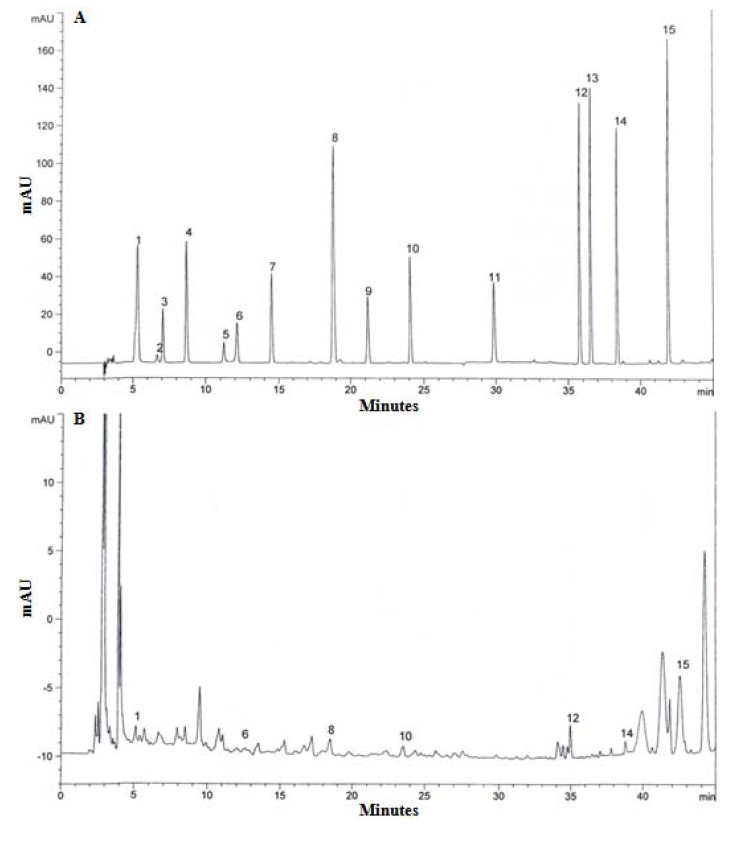
High performance liquid chromatography of phenolic compounds. A, Standard mixture of 15 phenolic compounds; B, *Lentinus lepideus* extract. 1, gallic acid; 2, pyrogallol; 3, homogentisic acid; 4, protocatechuic acid; 5, (+) catechin; 6, chlorogenic acid; 7, caffeic acid; 8, vanillin; 9, ferulic acid; 10, naringin; 11, resveratrol; 12, naringenin; 13, hesperetin; 14, formononetin; 15, biochanin- A.

Gallic acid, pyrogallol, homogentisic acid, protocatechuic acid, (+)-catechin, chlorogenic acid, caffeic acid, vanillin, ferulic acid, naringin, resveratrol, naringenin, hesperetin, formononetin, biochanin-A were used as standards for the detection of phenolic compounds from the extract of *L. lepideus*. Seven phenolic compounds, gallic acid, chlorogenic acid, vanillin, naringin, naringenin, formononetin, biochanin-A were detected from the acetonitrile and hydrochloric acid (5:1) solvent extract (Figure 3). The highest concentration of phenolic compound was recorded for chlorogenic acid (35 μg/g), followed by gallic acid (32 μg/g), biochanin-A (22 μg/g), vanillin (17 μg/g), naringin (15 μg/g), naringenin (15 μg/g), and formononetin (12 μg/g). These findings are comparable to the previous studies on *P. ostreatus* [17] in which six phenolic compounds – gallic acid, homogentisic acid, protocatechuic acid, chlorogenic acid, naringin, and myricetin – were identified and the total concentration of phenolic compounds was 90 μg/g.

Mushroom species also contain different types of phenolic compounds in varying numbers, ranging from three to 15, while gallic acid is reported as a common phenolic compound in mushrooms. Thus, the content of phenolic compounds could be used as an important indicator of antioxidant capacity. Several reports have convincingly shown a close relationship between phenolic content and antioxidant activity [18]. Mushroom extracts, which have high levels of phenolic compounds composed of one or more aromatic rings bearing one or more hydroxyl groups, can exhibit extensive free radical-scavenging activities as hydrogen donors or electron-donating agents, as well as metal ion-chelating properties. The greater numbers of hydroxyl groups in the phenolics could account for their higher antioxidant activity [19].

### 2.6. Xanthine oxidase inhibitory activity 

**Figure 4:**
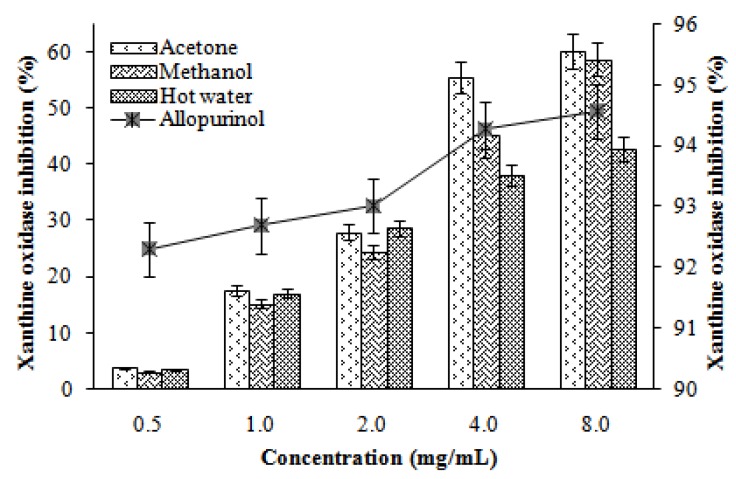
Xanthine oxidase inhibitory activity of various extracts from the fruiting bodies of *Lentinus lepideus*. Values expressed as means ± SD (n = 3).

Xanthine oxidase inhibitory activities of various extracts of *L. lepideus* increased with increasing concentration. At 0.5–8.0 mg/mL, the xanthine oxidase inhibition of acetone, methanol, and hot water extracts ranged from 3.82–60.25, 3.07–58.76, and 3.56–42.82%, respectively. However, at the same concentrations, allopurinol showed excellent xanthine oxidase inhibitory activity (92.31–94.58%, Figure 4). The results indicated that the acetone and methanol extracts showed good activity, while the hot water extract showed moderate activity at the concentration tested. However, at higher extraction doses, xanthine oxidase would be significantly inhibited.

Flavonoids are a group of polyphenolic compounds, which have been reported to possess xanthine oxidase inhibitory activity [20]. Hence, the presence of phenolic and flavonoid content in the extract would have contributed towards xanthine oxidase inhibition.

### 2.7. Tyrosinase inhibition 

**Figure 5:**
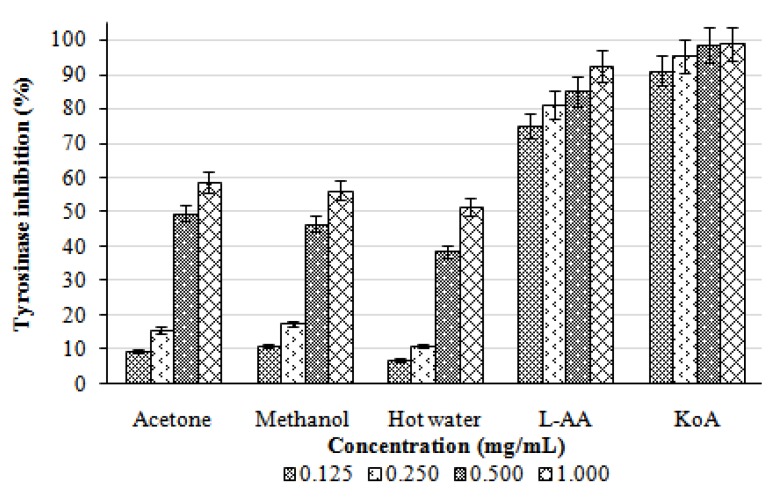
Tyrosinase inhibitory activity of various extracts from the fruiting bodies of *Lentinus lepideus*. Values expressed as means ± SD (n = 3); L-AA, L-ascorbic acid; KoA, kojic acid.

Tyrosinase inhibitory activities of the acetone, methanol, and hot water extracts from the fruiting bodies of *L. lepideus* increased with increasing concentration. At 0.125–1.0 mg/mL, the tyrosinase inhibition of acetonic, methanolic, and hot water extracts ranged from 9.71–58.84, 11.23–56.22, and 6.97–51.52%, respectively (Figure 5). The results indicated that the acetone and methanol extracts showed good activity, while the hot water extract showed only moderate activity at the concentration tested. At 0.125–1.0 mg/mL, the positive controls, L-ascorbic acid and kojic acid showed excellent tyrosinase inhibitory activities of 75.12–92.74% and 91.23–99.00%, respectively.

The tyrosinase inhibition ability might depend on the hydroxyl groups of the phenolic compounds of the mushroom extracts that could form a hydrogen bond to the active site of the enzyme, leading to a lower enzymatic activity. Some tyrosinase inhibitors act through hydroxyl groups that bind to the active site on tyrosinase, resulting in steric hindrance or changed conformation [21]. Gallic acid, (-)-epicatechin, procyanidin B2 and (-)-epicatechin-3-gallate, identified in mushrooms, all proved to be effective inhibitors of tyrosinase activity, as reported by many other authors [22,23]. The antioxidant activity may also be one of the important mechanisms for tyrosinase inhibitory activity.

## 3. Experimental 

### 3.1. Chemicals and reagents

β-Carotene, linoleic acid, chloroform, polyoxyethylene sorbitan monopalmitate (Tween40), butylated hydroxytoluene (BHT), α-tocopherol (TOC), 1,1-diphenyl-2-picrylhydrazyl (DPPH), L-ascorbic acid, potassium ferricyanide, trichloroacetic acid, ferrous chloride, ferric chloride, ferrozine, Folin-Ciocalteu reagent, gallic acid, methanol, 3,4-dihydroxy-L-phenylalanine (L-DOPA), xanthine, allopurinol, mushroom tyrosinase, and dimethyl sulfoxide (DMSO) were obtained from Sigma-Aldrich (St. Louis, MO, USA). All chemicals and solvents were used as HPLC or analytical grade.

### 3.2. Mushroom and extraction 

Fresh and mature fruiting bodies of *L. lepideus* were obtained from Mushroom Research Institute of Gyeonggi Province in Korea. A pure culture was deposited in Culture Collection and DNA Bank of Mushroom (CCDBM), Division of Life Sciences, University of Incheon, Korea and acquired accession number, IUM-4459. Fruiting bodies were dried with hot air at 40 °C for 48 h and finely pulverized. Five grams of powdered samples were extracted with 100 mL of 60% acetone and 80% methanol with stirring at 150 rpm for 24 h at 25 °C to obtain acetonic and methanolic extracts. The mixture was filtered through two layer of Whatman No. 1 filter paper. The same quantity of sample was boiled at 100 °C for 3 h with 100 mL deionized distilled water to obtain a hot water extract. The mixture was cooled to room temperature and filtered through Whatman no. 1 filter paper. The residues were then extracted with two additional 100 mL aliquots of acetone, methanol, and deionized water, as described above. The combined extracts were evaporated with a rotary evaporator (Eyela, Saitama, Japan) at 40 °C, and the remaining solvent was removed with a freeze-drier (Optizen, Daejeon, Korea). The yields from the acetonic, methanolic and hot water extracts of *L. lepideus* were 27.24, 20.24 and 23.80% (w/w), respectively.

### 3.3. Antioxidant activity by β-carotene-linoleic acid 

Antioxidant activity was determined by measuring the inhibition of volatile organic compounds and the conjugated diene hydroperoxides arising from linoleic acid oxidation [24]. A stock solution of a β-carotene-linoleic acid mixture was prepared as following: 0.5 mg β-carotene was dissolved in 1 mL of chloroform, and 25 μL of linoleic acid and 200 mg Tween 40 was added. The chloroform was removed completely using a vacuum evaporator. Then, 100 mL of oxygenated distilled water was added with vigorous shaking; 2.5 mL of this reaction mixture was dispensed to test tubes, 0.5 mL of various concentrations (0.5–20.0 mg/mL) of the extracts in methanol was added, and the reaction mixture was incubated for up to 2 h at 50 °C. The same procedure was repeated with the positive control BHT and TOC, and a blank. After the incubation, the absorbance of the mixtures was measured at 490 nm using a spectrophotometer (Optizen POP; Mecasys Co. Ltd., Daejeon, Korea). The absorbance was measured until the β-carotene color disappeared. The β-carotene bleaching rate (R) was calculated according to Eq. (1):

R = ln (a/b)/ t
(1)

where, ln = natural log, a = absorbance at time t (0), b = absorbance at time t (120 min). The antioxidant activity (AA) was calculated as the percent inhibition relative to the control using Eq. (2):

AA = [(R_control_ − R_sample_) /R_control_] × 100 (2)

Antioxidant activities of the extracts were compared with those of BHT and TOC at 0.5 mg/mL and a blank consisting of 0.5 mL methanol.

### 3.4. Reducing power 

Reducing power was determined according to the method of Gulcin *et al.* [25]. Each extract (1–8 mg/mL) in methanol (2.5 mL) was mixed with 2.5 mL of 200 mM sodium phosphate buffer (pH 6.6) and 2.5 mL of 1% potassium ferricynide, and the mixture was incubated at 50 °C for 20 min. Then, 2.5 mL of 10% trichloroacetic acid was added, and the mixture was centrifuged at 200 × g (6K 15; Sigma, Mannchein, Germany) for 10 min. The upper layer (2.5 mL) was mixed with 2.5 mL of deionized water and 0.5 mL of 0.1% ferric chloride. Finally, the absorbance was measured at 700 nm against a blank. BHT and TOC were used as positive control.

### 3.5. Scavenging effect on 1,1-diphenyl-2-picrylhydrazyl radicals 

The hydrogen atoms or electrons donation ability of the corresponding extracts and some pure compounds were measured from the bleaching of purple colored DPPH methanol solution [26]. Four mL of various concentrations (0.125–2.0 mg/mL) of the extracts in methanol was added to 1 mL of DPPH radical solution in methanol (final concentration of DPPH was 0.2 mM). The mixture was shaken vigorously and allowed to stand for 30 min, and the absorbance of the resulting solution was measured at 517 nm using a spectrophotometer. Inhibition of the DPPH free radical in percent (I %) was calculated as:

I % = [(A_control_ − A_sample_)/A_control_] × 100

where, A_control_ is the absorbance of the control reaction (containing all reagents except the test compound), and A_sample_ is the absorbance of the test compound. BHT, TOC, and L-ascorbic acid were used as positive controls.

### 3.6. Chelating effects on ferrous ions 

The chelating effect was determined according to the method of Dinis *et al.* [27]. Briefly, 2 mL of various concentrations (0.063–1.0 mg/mL) of the extracts in methanol was added to a solution of 2 mM FeCl_2_ (0.05 mL). The reaction was initiated by adding 5 mM ferrozine (0.2 mL). Total volume was adjusted to 5 mL with methanol, and the mixture was shaken vigorously and left at room temperature for 10 min. The absorbance of the solution was measured spectrophotometrically at 562 nm. The inhibition percentage of the ferrozine-Fe^2+^ complex formation was calculated using the following formula:

Metal chelating effect (%) = [(A_control_ − A_sample_)/A_control_] × 100

where, A_control_ is the absorbance of the control (control contained FeCl_2_ and ferrozine; complex formation molecules), and A_sample_ is the absorbance of the test compound. BHT and TOC were used as positive controls.

### 3.7. Analysis of phenolic compounds 

Fifteen standard phenolic compounds, including gallic acid, pyrogallol, homogentisic acid, protocatechuic acid, (+) catechin, chlorogenic acid, caffeic acid, vanillin, ferulic acid, naringin, resveratrol, naringenin, hesperetin, formononetin, biochanin-A were purchased from Sigma Aldrich and used for calibration curves. The standard stock solutions (50, 100, 250, and 500 ppm) were made with DMSO. Sample compounds were identified based on retention times of authentic standards and were quantified by comparing their peak areas with those of the standard curves.

Sample preparation for the phenolic compound analysis followed Kim *et al.* [28]. Two grams of dried mushroom powder were mixed with 10 mL of acetonitrile and 2 mL of 0.1 N hydrochloric acid and stirred 150 rpm for 2 h at room temperature. The suspension was filtered through Whatman no. 42 filter paper. The extract was freeze-dried, and the residues were redissolved in 10 mL of 80% aqueous methanol (HPLC grade) and filtered through a 0.45 μM nylon membrane filter (Titan, Rockwood, TN, USA). The 20 μL filtrate was loaded onto an Agilent-1100 series liquid chromatography HPLC system (Agilent Technologies, Waldbronn, Germany). Separation was achieved on a 250 nm × 4.6 mm i.d., 5 μM, YMC-Pack ODS AM (YMC, Kyoto, Japan) column. The mobile phase was distilled water with 0.1% glacial acetic acid (solvent A) and acetonitrile with 0.1% glacial acetic acid (solvent B). The gradient was 0 min, 92% A; 0–2 min, 90% A; 2–27 min, 70% A; 27–50 min, 10% A; 50–51 min, 0% A; 51–60 min, 0% A; 60–63 min, 92% A. The run time was 60 min using a flow rate of 1 mL/min Detection was performed with a diode array detector at a wavelength of 280 nm.

### 3.8. Xanthine oxidase inhibition

*In vitro* xanthine oxidase inhibitory activity of various extracts from the fruiting bodies of *L. lepideus* was assayed spectrophotometrically under aerobic conditions using xanthine as the substrate [29]. The assay mixture consisted of 1 mL extract of the different concentrations (0.5–8.0 mg/mL), 2.9 mL of phosphate buffer (pH 7.5), and 0.1 mL of xanthine oxidase enzyme solution (0.1 units/mL in phosphate buffer, pH 7.5), which was prepared immediately before use. After pre incubation at 25 °C for 15 min, the reaction was initiated by the addition of 2 mL of the substrate solution (150 μM xanthine in the same buffer). The assay mixture was incubated at 25 °C for 30 min. The reaction was then stopped by the addition of 1 mL of 1N hydrochloric acid and the absorbance was measured at 290 nm using a spectrophotometer. Different concentrations of the extracts were dissolved in DMSO and the final concentration of DMSO was 5%, which did not affect the enzyme assay. Allopurinol (0.5–8.0 mg/mL), a known inhibitor of XO, was used as positive control. One unit of XO is defined as the amount of enzyme required to produce 1 mmol of uric acid/min at 25 °C. Xanthine oxidase inhibitory activity was expressed as the percentage inhibition of XO in the above assay system calculated as:

Inhibition (%) = [(A − B) − (C − D)/(A − B)] × 100

where A is the activity of the enzyme without the extraction, B is the control of A without the extraction and enzyme; C and D are the activities of the extraction with and without XO, respectively.

### 3.9. Tyrosinase inhibition 

Tyrosinase inhibition activity was determined using the modified dopachrome method with L-DOPA as substrate [30]. A 96-well microtitre plate was used to measure absorbance at 475 nm with 700 nm as reference. Extract fraction were dissolved in 50% DMSO. Each well contained 40 μL of sample with 80 μL of phosphate buffer (0.1 M, pH 6.8), 40 μL of tyrosinase (31 units/mL), and 40 μL of L-DOPA (2.5 mM). The mixture was incubated for 10 min at 37 °C, and absorbance was measured at 475 nm using a UVM 340 microplate reader (Asys, Eugendrof, Austria). Each sample was accompanied by a blank containing all components except L-DOPA. L-Ascorbic acid and kojic acid were used as positive controls. The results were compared with a control consisting of 50% DMSO in place of the sample. The percentage of tyrosinase inhibition was calculated as follows:

[(A_control_ − A_sample_)/A_control_] × 100

### 3.10. Statistical analysis 

Data were expressed as means ± standard deviations of three replicate determinations and were analyzed by SPSS V.13 (SPSS Inc., Chicago, IL, USA). One way analysis of variance and Duncan’s new multiple-range test were used to determine the differences among the means.

## 4. Conclusions 

Wild edible mushroom, *L. lepideus* is relatively new and commercially available in Korea. This study is the first report about the antioxidant and antityrosinase activities and the detection of phenolic compounds of *L. lepideus*. The study showed that *L. lepideus* had higher chelating effects on ferrous ions compared with those of BHT and TOC. The high phenolic content exhibited good antioxidant and antityrosinase activities. Furthermore seven phenolic compounds were detected in the fruiting bodies of *L. lepideus*. On the basis of the results, it is suggested that *L. lepideus* can be used as a comprehensible source of natural antioxidants for the nourishment. 

## Figures and Tables

**Table 1:** Antioxidant activity against β-carotene-linoleic acid of different concentrations of various extracts from the fruiting bodies of *Lentinus lepideus*.

Solvent and control	Sample concentration (mg/mL)			
	0.5	2.0	8.0	20.0
Acetone	68.48 ± 0.59 c	86.74 ± 0.23 a	92.63 ± 0.18 b	95.52 ± 0.37 a
Methanol	59.69 ± 0.71 d	85.03 ± 0.67 a,b	91.23 ± 0.53 b	95.42 ± 0.02 a
Hot water	68.73 ± 0.32 c	88.12 ± 0.99 a	96.32 ± 0.25 a	96.33 ± 0.86 a
BHT	95.21 ± 0.17 a	-	-	-
TOC	96.02 ± 0.18 a	-	-	-

Values expressed as means ± SD (n = 3); -, not analyzed; BHT, butylated hydroxytoluene; TOC, α-tocopherol; Means with different letters within a column are significantly different (*p* ≤ 0.05).

**Table 2:** Reducing power of different concentrations of various extracts from the fruiting bodies of *Lentinus lepideus*.

Solvent and control	Sample concentration (mg/mL)			
	1.0	2.0	4.0	8.0
Acetone	0.31 ± 0.03 e	0.46 ± 0.03 ab	0.73 ± 0.04 b	1.12 ± 0.05 ab
Methanol	0.36 ± 0.07 e	0.53 ± 0.11 a	0.82 ± 0.11 a	1.21 ± 0.31 a
Hot water	0.32 ± 0.26 e	0.42 ± 0.22 b	0.64 ± 0.19 c	1.10 ± 0.11 ab
BHT	3.21 ± 0.49 a	-	-	-
TOC	2.16 ± 0.32 b	-	-	-

Values expressed as means ± SD (n = 3); -, not analyzed; BHT, butylated hydroxytoluene; TOC, α-tocopherol; Means with different letters within a column are significantly different (*p* ≤ 0.05).

## References

[1] Hibbett D.S., Vilgalys R. (1993). Phylogenetic relationships of *Lentinus* (Basidiomycotina) inferred from molecular and morphological characters. Syst. Bot..

[2] Mattila P., Konko K., Eurola M., Pihlava J.M., Astola J., Vahteristo L., Hietaniemi V., Kumpulainen J., Valtonen M., Piironen V. (2001). Contents of vitamins, mineral elements, and some polyphenolic compounds in cultivated mushrooms. J. Agric. Food Chem..

[3] Cheung L.M., Cheung P.C., Ooi V.E. (2003). Antioxidant activity and total phenolics of edible mushroom extracts. Food Chem..

[4] Fukushoma M., Ohashi T., Fujiwara Y., Sonoyama K., Nakano M. (2001). Cholesterol-lowering effects of maitake (*Grifola frondosa*) fiber, shiitake (*Lentinus edodes*) fiber, enokitake (*Flammulina velutipes*) fiber in rats. Exp. Biol. Med..

[5] Velioglu Y.S., Mazza G., Gao L., Oomah B.D. (1998). Antioxidant activity and total phenolics in selected fruits, vegetables, and grain products. J. Agric. Food Chem..

[6] Turkoglu A., Duru M.E., Mercan N., Kivrak I., Gezer K. (2007). Antioxidant and antimicrobial activities of *Laetiporus sulphureus* (Bull.) Murrill. Food Chem..

[7] Zhou C.X., Kong L.D., Ye W.C., Cheng C.H., Tan R.X. (2001). Inhibition of xanthine and monoamine oxidases by stillbenoids from *Veratrum taliense*. Planta Med..

[8] Chang W.S., Lee Y.J., Lu F.J., Chaing H.C. (1993). Inhibitory effects of flavonoids on xanthine oxidase. Anticancer Res..

[9] Hearing V.J., Jimenez M. (1987). Mammalian tyrosinase- the critical regulatory control point in melanocyte pigmentation. Int. J. Biochem..

[10] Barros L., Ferreira M.J., Queiros B., Ferreira I.C., Bapista P. (2007). Total phenols, ascorbic acid, β-carotene and lycopene in Portuguese wild edible mushrooms and their antioxidant activities. Food Chem..

[11] Lee Y.L., Huang G.W., Liang Z.C., Mau J.L. (2007). Antioxidant properties of three extracts from *Pleurotus citrinopileatus*. LWT-Food Sci. Technol..

[12] Shimada K., Fujikawa K., Yahara K., Nakamura T. (1992). Antioxidative properties of xanthan on the autoxidation of soybean oil in cyclodextrin emulsion. J. Agric. Food Chem..

[13] Herraiz T., Galisteo J., Chamorro C. (2003). L-tryptophan reacts with naturally occurring and food-occurring phenolic aldehydes to give phenolic tetrahydro-β-caroline alkaloids: Activity as antioxidants and free radical scavengers. J. Agric. Food Chem..

[14] Tsai S.Y., Huang S.J., Mau J.L. (2006). Antioxidant properties of hot water extracts from *Agrocybe cylindracea*. Food Chem..

[15] Lee Y.L., Yen M.T., Mau J.L. (2007). Antioxidant properties of various extracts from *Hypsizigus marmoreus*. Food Chem..

[16] Yamaguchi R., Tatsumi Y., Asano M., Kato K., Ueno Y. (1988). Effect of metal salts and fructose on the autoxidation of methyl linoleate in emulsions. Agric. Biol. Chem..

[17] Kim M.Y., Seguin P., Ahn J.K., Kim J.J., Chun S.C., Kim E.H., Seo S.H., Kang E.Y., Kim S.L., Park Y.J., Ro H.M., Chung I.M. (2008). Phenolic compound concentration and antioxidant activities of edible and medicinal mushrooms from Korea. J. Agric. Food Chem..

[18] Pan Y., Wang K., Huang S., Wang H., Mu X., He C., Ji X., Zhang J., Huang F. (2008). Antioxidant activity of microwave-assisted extract of longan (*Dimocarpus longan* Lour.) peel. Food Chem..

[19] Prasad K.N., Divakar S., Shivamurthy G.R., Aradhya S.M. (2005). Isolation of a free radical scavenging antioxidant from water spinach (*Ipomoea aquatica* Forsk). J. Sci. Food Agric..

[20] Costantino L., Albasini A., Rastelli G., Benvenuti S. (1992). Activity of polyphenolic crude extracts as scavengers of superoxide radicals and inhibitors of xanthine oxidase. Planta Med..

[21] Baek H.S., Rho H.S., Yoo J.W., Ahn S.M., Lee J.Y., Lee J., Kim M.K., Kim D.H., Chang I.S. (2008). The inhibitory effect of new hydroxamic acid derivatives on melanogenesis. Bull. Korean Chem. Soc..

[22] Kubo I., Chen Q.X., Nihei K.I. (2003). Molecular design of antibrowning agents: antioxidative tyrosinase inhibitors. Food Chem..

[23] Momtaz S., Mapunya B.M., Houghton P.J., Edgerly C., Hussein A., Naidoo S., Lall N. (2008). Tyrosinase inhibition by extracts and constituents of *Sideroxylon inerme* L. stem bark, used in South Africa for skin lightening. J. Ethnopharmacol..

[24] Dapkevicius A., Venskutonis R., van Beek T.A., Linssen J.P. (1998). Antioxidant activity of extracts obtained by different isolation procedures from some aromatic herbs grown in Lithuania. J. Sci. Food Agric..

[25] Gulcin I., Buyukokuroglu M.E., Oktay M., Kufrevioglu O.I. (2003). Antioxidant and analgesic activities of turpentine of *Pinus nigra* Arn. subsp. *pallsiana* (Lamb.) Holmboe. J. Ethnopharmacol..

[26] Cuendet M., Hostettmann K., Potterat O., Dyatmiko W. (1997). Iridoid glucosides with free radical scavenging properties from *Fagraea blumei*. Helv. Chim. Acta.

[27] Dinis T.C., Madeira V.M., Almeida L.M. (1994). Action of phenolic derivatives (acetaminophen, salicylate, and 5-amino salicylate) as inhibitors of membrane lipid peroxidation and as peroxyl radical scavengers. Arch. Biochem. Biophys..

[28] Kim E.H., Kim S.H., Chung J.I., Chi H.Y., Kim J.A., Chung I.M. (2006). Analysis phenolic compounds and isoflavones in soybean seeds (*Glycine max* (L.) Merill) and sprouts grown under different conditions. Eur. Food Res. Technol..

[29] Owen P.L., Johns T. (1999). Xanthine oxidase inhibitory activity of North-Eastern North American plant remedies used for gout. J. Ethnopharmacol..

[30] Masuda T., Yamashita D., Takeda Y., Yonemori S. (2005). Screening for tyrosinase inhibitors among extracts of seashore plants and identification of potent inhibitors from *Garcinia subelliptica*. Biosci. Biotechnol. Biochem..

